# Synthesis of coaxial nanotubes of polyaniline and poly(hydroxyethyl methacrylate) by oxidative/initiated chemical vapor deposition

**DOI:** 10.3762/bjnano.8.89

**Published:** 2017-04-18

**Authors:** Alper Balkan, Efe Armagan, Gozde Ozaydin Ince

**Affiliations:** 1 Materials Science and Nanoengineering Program, Faculty of Engineering and Natural Sciences, Sabanci University, Tuzla, Istanbul 34956, Turkey; 2 Nanotechnology Application Center, Sabanci University, Orhanli, Tuzla, Istanbul 34956, Turkey

**Keywords:** coaxial nanotubes, humidity sensors, initiated chemical vapor deposition, oxidative chemical vapor deposition, polyaniline

## Abstract

Vapor-phase synthesis techniques of polymeric nanostructures offer unique advantages over conventional, solution-based techniques because of their solventless nature. In this work, we report the fabrication of coaxial polymer nanotubes using two different chemical vapor deposition methods. The fabrication process involves the deposition of an outer layer of the conductive polyaniline (PANI) by oxidative chemical vapor deposition, followed by the deposition of the inner layer of poly(2-hydroxyethyl methacrylate) (pHEMA) hydrogel by initiated chemical vapor deposition. The vapor-phase techniques allowed for fine-tuning of the thickness of the individual layers, keeping the functionalities of the polymers intact. The response of the single components and the coaxial nanotubes to changes in humidity was investigated for potential humidity sensor applications. For single-component conductive PANI nanotubes, the resistance changed parabolically with relative humidity because of competing effects of doping and swelling of the PANI polymer under humid conditions. Introducing a hydrogel inner layer increased the overall resistance, and enhanced swelling, which caused the resistance to continuously increase with relative humidity.

## Introduction

In recent years, with the advances in nanotechnology, the use of nanostructured materials has become widespread in various applications, such as biotechnology [1–2], food industry [3–4], sensors [5] or photovoltaics [6]. Polymeric nanostructures have attained special interest because of their prominent advantages, such as cost-effectiveness, ease of fabrication and biocompatibility making these nanostructures applicable in different areas [7–8]. Among these, the conducting polymer (CP) nanostructures, such as nanowires, nanorods, nanotubes or nanospheres have been extensively studied through solution-based techniques, such as chemical polymerization [9–11] or electrochemical polymerization [12–14] for applications in light emitting diodes [15], photovoltaic cells [16–17], supercapacitors [18], sensors [19] and drug delivery [20]. During synthesis of these nanostructures, the use of solvents is a major drawback for homogeneity and conformal coatings, especially on high-aspect-ratio templates, due to wetting effect and surface tension. Thus, vapor-phase polymerization techniques have emerged for the deposition of conducting polymers that facilitate the fabrication of conformal polymeric structures [21–22].

Polyaniline (PANI) is one of the well-known conducting polymers with applications in supercapacitors [18], sensors [23], solar cells [24] and membranes [25] because of its great thermal and environmental stability, ease of synthesis, excellent conductivity, cost-effectiveness and redox-tunability [26–29]. One of the original aspects of PANI is that its electrical conductivity can be tuned through oxidation and protonation steps. Depending on the oxidation level, PANI can exist in three different states: leucomeraldine base (fully reduced), emeraldine base (half-oxidized) and pernigraniline base (fully oxidized). However, only the emeraldine salt which is the protonated form of emeraldine has a good conductivity of 1–130 S/cm [30–31].

PANI is a good candidate material for sensor applications due to the change of oxidation/reduction level in response to changes in the environmental conditions, which, in turn, affect the electrical conductivity [32]. However, studies on PANI as humidity sensors are very limited. Zeng et al. [33] studied the resistance change of PANI nanofibers depending on the humidity level. They observed that the resistance changed parabolically as the humidity of the environment increased, and a minimum value for the resistance at a certain humidity level existed. The parabolic behavior with the same resistance readings for two different humidity levels is problematic for sensor applications, making the sensor unreliable. In order to modify this parabolic behavior, Parvatikar et al. [34] fabricated PANI/CeO_2_ composites, whose resistance values decreased linearly as humidity increased due to charge transfer between CeO_2_ and PANI. However, incorporating CeO_2_ in the polymer decreases flexibility and increases the overall electrical resistance, which may limit the range of applications. Lin et al. [35] fabricated electrospun PANI nanofibers and introduced hydrophilic poly(ethylene oxide) and hydrophobic poly(vinyl butyral) into PANI to tune the sensitivity towards humidity. It was observed that increasing the fraction of the hydrophilic material within the sensor decreased resistance, whereas increasing the hydrophobicity resulted in higher resistance.

Our work here demonstrates the advantages of fabricating PANI nanotubes in combination with a hydrophilic material, namely poly(2-hydroxyethyl methacrylate) (pHEMA), enabling PANI to be used in humidity sensors with higher humidity sensitivity due to the open-mouth structure and the high surface area of the nanotubes. Furthermore, fabricating conductive nanotubes using templates with mesoscopic pores resulted in the alignment of polymer chains parallel to the tube axis, increasing conductivity above that of nonaligned films [36]. In this study, the fabrication of PANI nanotubes and PANI/pHEMA coaxial nanotubes were done via oxidative chemical vapor deposition (oCVD) and initiated chemical vapor deposition (iCVD) to enhance the control and sensitivity level of humidity sensors. By using the vapor deposition method oCVD, we achieved conformal coatings of PANI, which allowed us to produce nanotubes with high purity and controlled wall thickness. Furthermore, the oxidation state of PANI could be controlled by varying the oxidant flowrate for the purpose of achieving conductive emeraldine salt. The oCVD technique is based on step-growth polymerization where the polymerization takes place directly on the surface of the substrate. The oxidant, either liquid [37] or solid [38], and the monomers are delivered into the vacuum system simultaneously, initiating the polymerization reaction on the surface. The key advantages of oCVD are good homogeneity, retention of polymer functional groups due to low reaction temperature (25–100 °C), adequate electrical conductivity for a wide range of applications and high-quality conformal CP thin films on various non-planar surfaces [39–41]. In this study, the vapor-phase oCVD and iCVD techniques were used to conformally coat the walls of the pores of anodized aluminium oxide (AAO) track-etch membranes. The ability to control the thickness with high sensitivity using these vapor phase techniques allowed to produce coaxial nanotubes. The response of these nanotubes to the changes in humidity could be tuned by introducing the hydrogel inner layer.

## Results and Discussion

**Figure 1:**
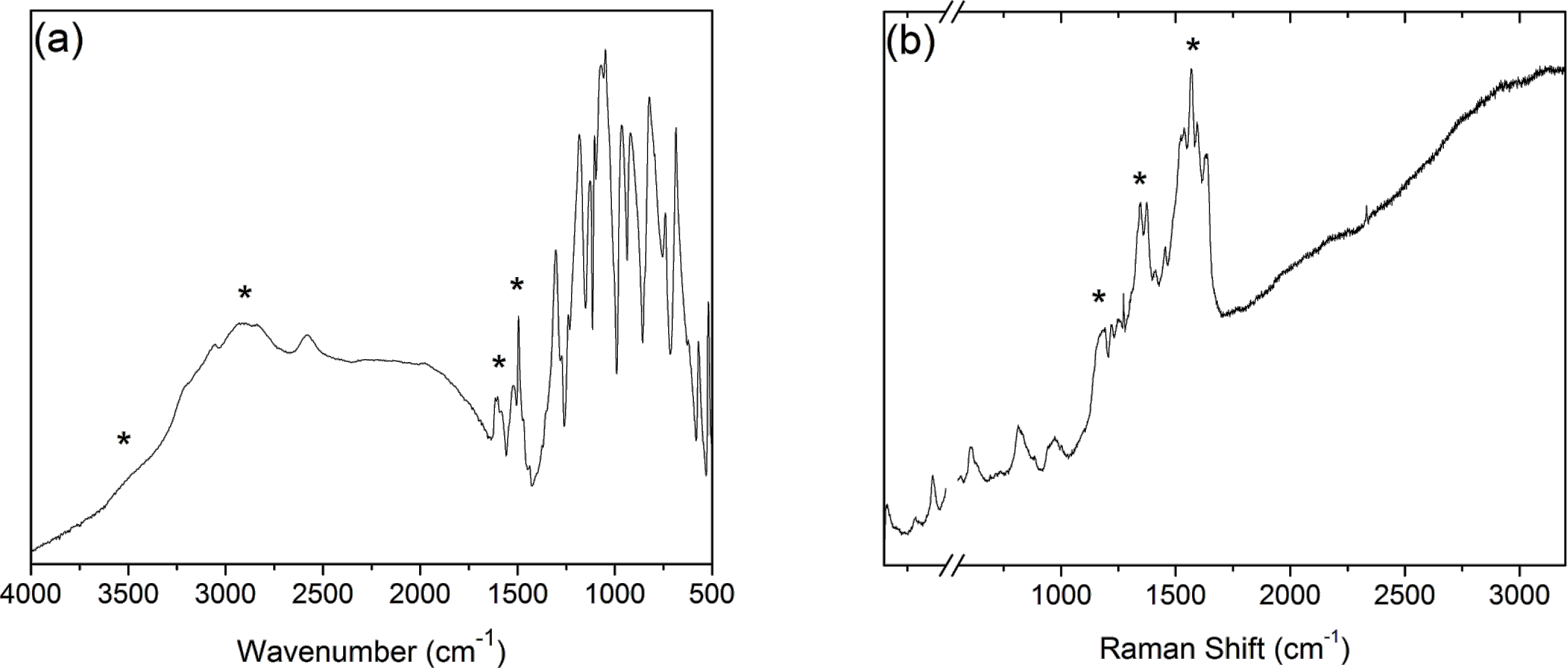
(a) FTIR spectra of PANI emeraldine thin film on a Si wafer. The peaks at 1590 and 1495 cm^−1^ correspond to stretching vibrations of the quinoid and benzenoid rings, respectively. (b) RAMAN spectra of PANI emeraldine thin film on a Si wafer. The peak at 1638 cm^−1^ corresponds to the C–C stretching vibrations in benzoid units, while the peaks due to the C=N and C=C stretching vibrations in quinoid units appear at 1458 and 1569 cm^−1^, respectively. Both the FTIR and RAMAN spectra confirm the polymerization of PANI.

The deposition of PANI films on a Si wafer was confirmed by Fourier-transform infrared (FTIR) analysis (Figure 1a). The broad peaks at 2850–3100 cm^−1^ and 3100–3600 cm^−1^ correspond to C–H and N–H stretching vibrations, respectively. The peak at 1590 cm^−1^ can be attributed to the quinoid ring stretching, while the peak at 1495 cm^−1^ is due to the benzenoid ring stretching [42].

A complementary structural analysis was performed with Raman spectroscopy (Figure 1b). The peak at 1193 cm^−1^ is due to C–H vibrations bending in benzoid units. The peaks at 1223 and 1272 cm^−1^ correspond to the bands related to amine groups. Between 1332 and 1376 cm^−1^, the vibrations of delocalized polaronic structures can be observed. The peaks at 1458 and 1569 cm^−1^ correspond to C=N and C=C stretching vibrations in quinoid units, respectively. At 1638 cm^−1^, the peak for C–C stretching vibrations in benzoid units is present. The results obtained are in good agreement with literature confirming the successful polymerization of PANI thin films [43].

**Figure 2:**
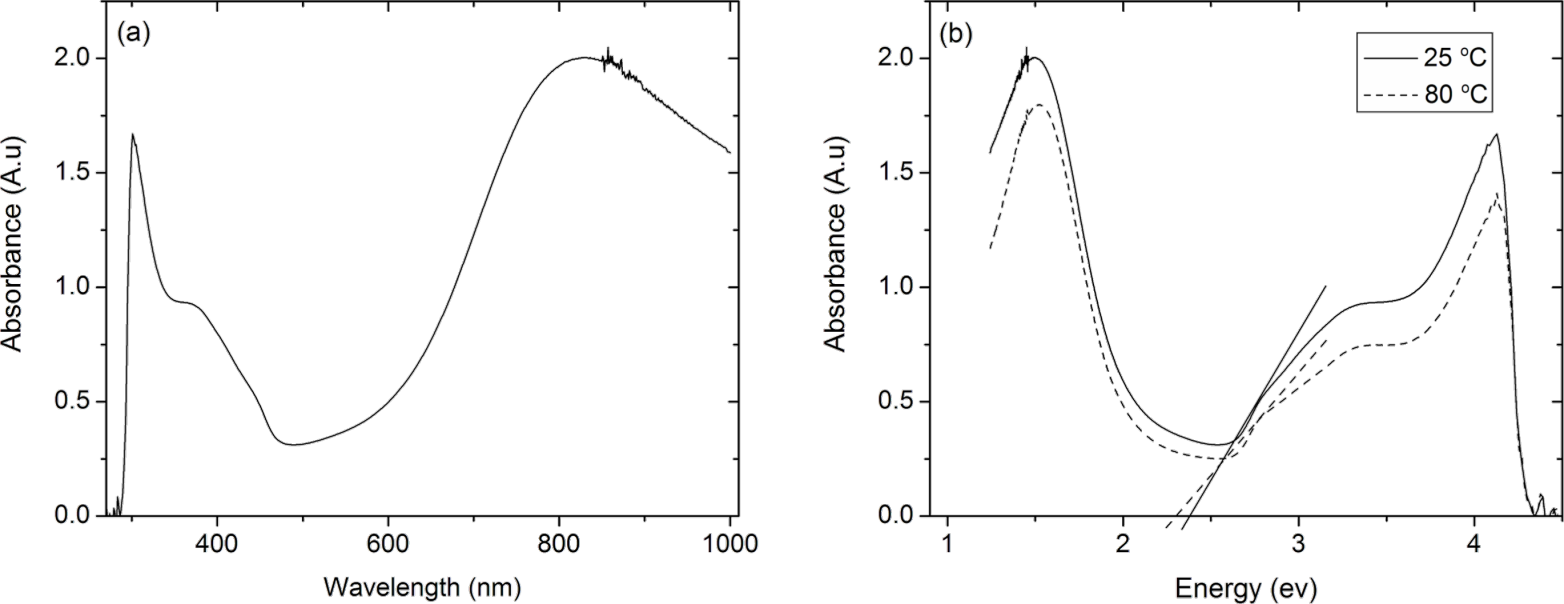
(a) UV–vis spectrum of as-deposited PANI thin films. Three characteristic peaks at 360, 430 and 796 nm indicate the formation of a polaron band transition. The peak at 430 nm due to the polaron–bipolaron band transition confirms the deposition of the emeraldine salt form of PANI. (b) UV–vis spectra of as-deposited and annealed PANI thin films. Bandgaps of 2.38 and 2.26 eV were calculated for the as-deposited and annealed samples, respectively, confirming the formation of the protonated emeraldine salt form of PANI.

In order to confirm the formation of the protonated emeraldine salt form of PANI, UV–vis analysis was performed on the thin film samples. Figure 2a shows the UV–vis spectra of the as-deposited PANI films with three characteristic peaks at 360, 430 and 796 nm, indicating the formation of a polaron band transition. Furthermore, the peak at 430 nm originates from polaron–bipolaron band transitions consistent with the emeraldine salt form of PANI [44].

The band gaps of both annealed (80 °C) and as-deposited PANI samples were found using the UV–vis spectra (Figure 2b). The band gap of PANI can be calculated from the wavelength of the polaron band excitation [45]. The onset of absorption of the polaron band excitation was used to find the band gap energies, *E*_g_, of both samples. The *E*_g_ of as-deposited and annealed samples were calculated as 2.38 and 2.26 eV, respectively. The slight decrease in the band gap with increasing annealing temperature is consistent with previous PANI studies. Joshi et al. reported that the band gap of PANI decreases as the annealing temperature increases up to 100 °C because of the formation of a new crystalline region and the rearrangement of the existing crystalline region [46]. However, annealing at temperatures above 100 °C initiates deformation and causes damage in the crystalline structure of PANI polymer chains resulting in the increase of the band gap energy.

**Figure 3:**
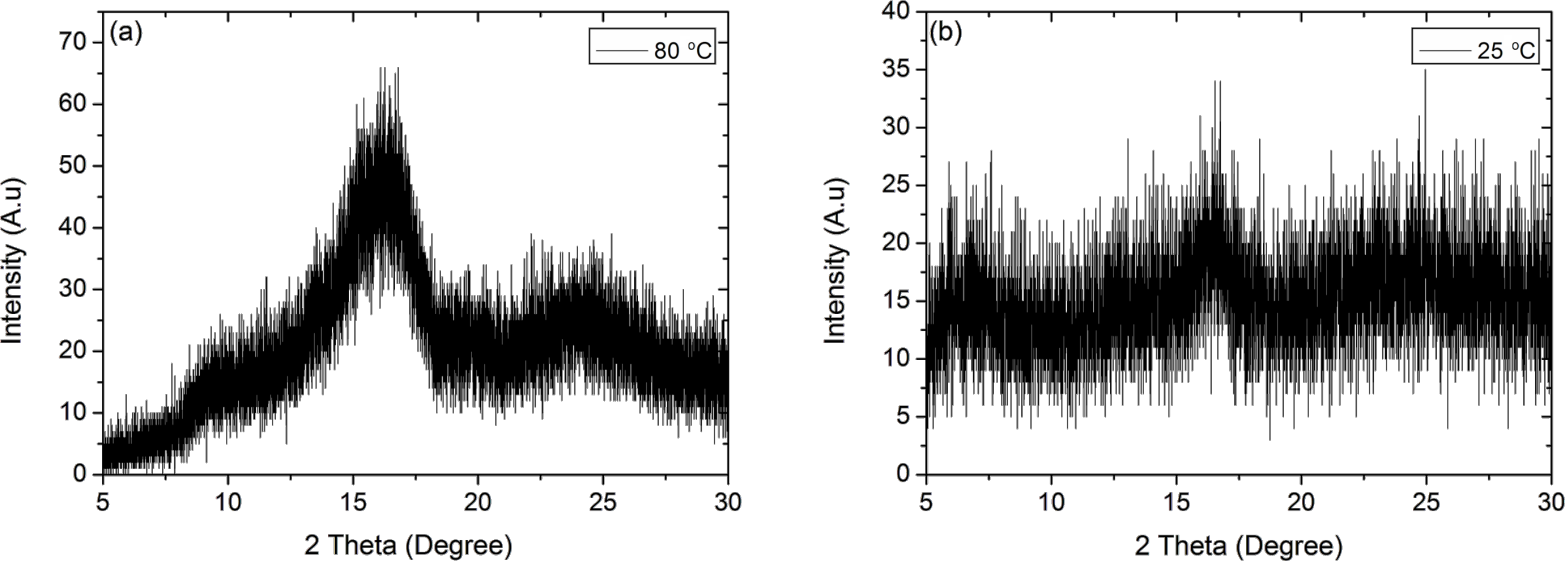
XRD spectra of (a) annealed and (b) as-deposited PANI thin films. After annealing at 80 °C for 4 h, characteristic peaks of PANI appear at 16° and 25°, which correspond to (011) and (200) planes, respectively, confirming the crystallinization of the polymer film upon annealing.

The crystallinity of the deposited films was studied by using XRD analysis (Figure 3). The spectra of the non-annealed, as-deposited samples did not show any distinct peaks, indicating the amorphous state of the films. However, after an annealing process at 80 °C for 4 h, the measurements revealed two peaks at the characteristic 2θ angles of PANI, 16° and 25°, which correspond to the (011) and (200) planes, respectively. These results are attributed to the reorganization of the chains during annealing to form crystalline regions. However, the broadness of the peaks indicate a low degree of crystallinity [47].

**Figure 4:**
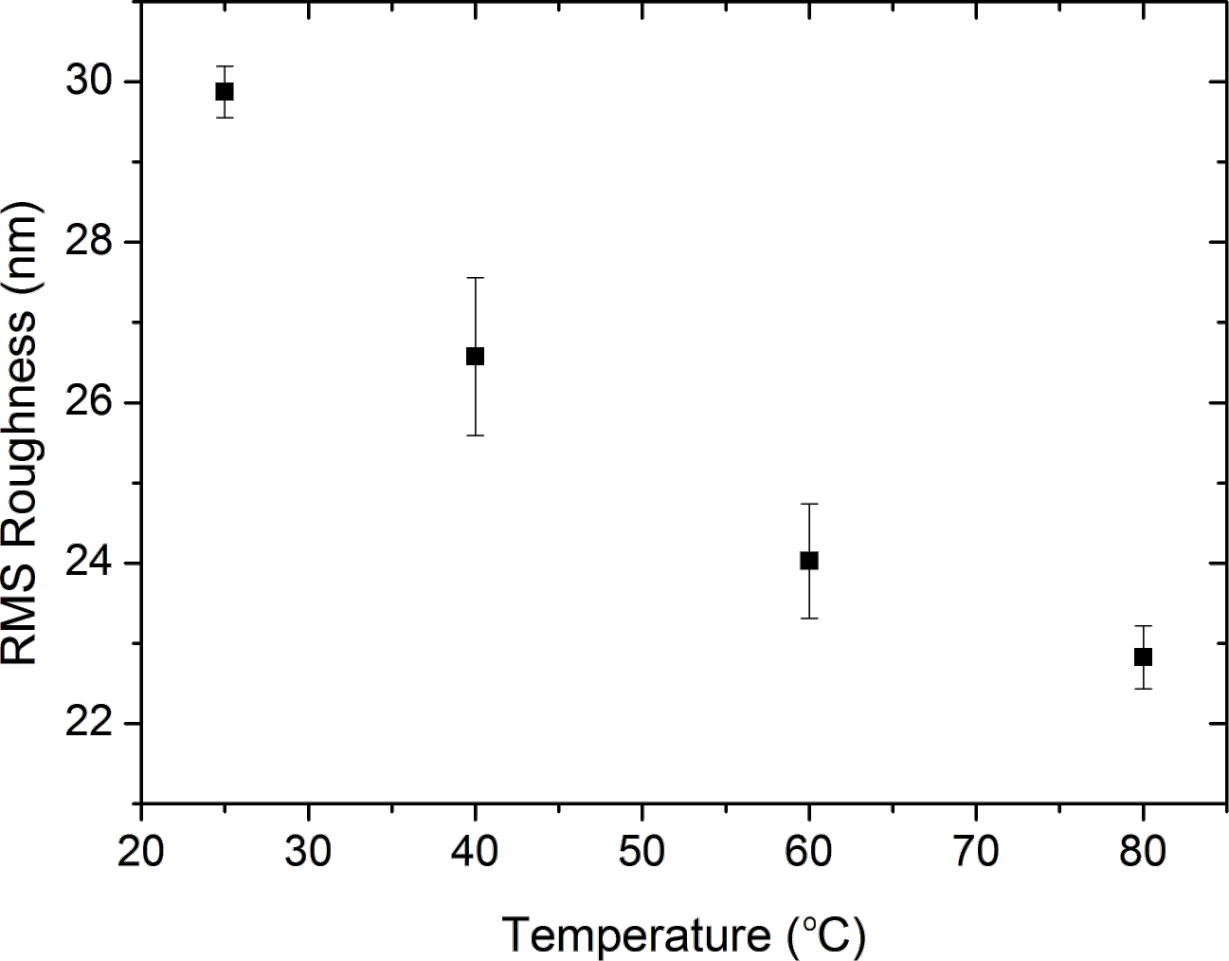
Surface roughness of PANI thin films annealed at 25, 40, 60 and 80 °C. The surface roughness of the polymer thin films decreases as a result of the increased crystallinity as the annealing temperature increases.

The surface morphology of the PANI thin films was examined by using AFM analysis (Figure 4). The RMS surface roughness of the as-deposited thin films of 350 nm thickness was measured as 30 nm on a flat substrate, and the roughness increased with film thickness. When the annealing temperature was increased, the RMS roughness of the PANI thin films decreased. The decrease in surface roughness with increasing temperatures can be explained by the rearrangement of amorphous part of polymer chains and formation of new crystalline regions on the surface that reduce the irregularity and increase the percentage of crystalline regions on the surface [48–49].

**Figure 5:**
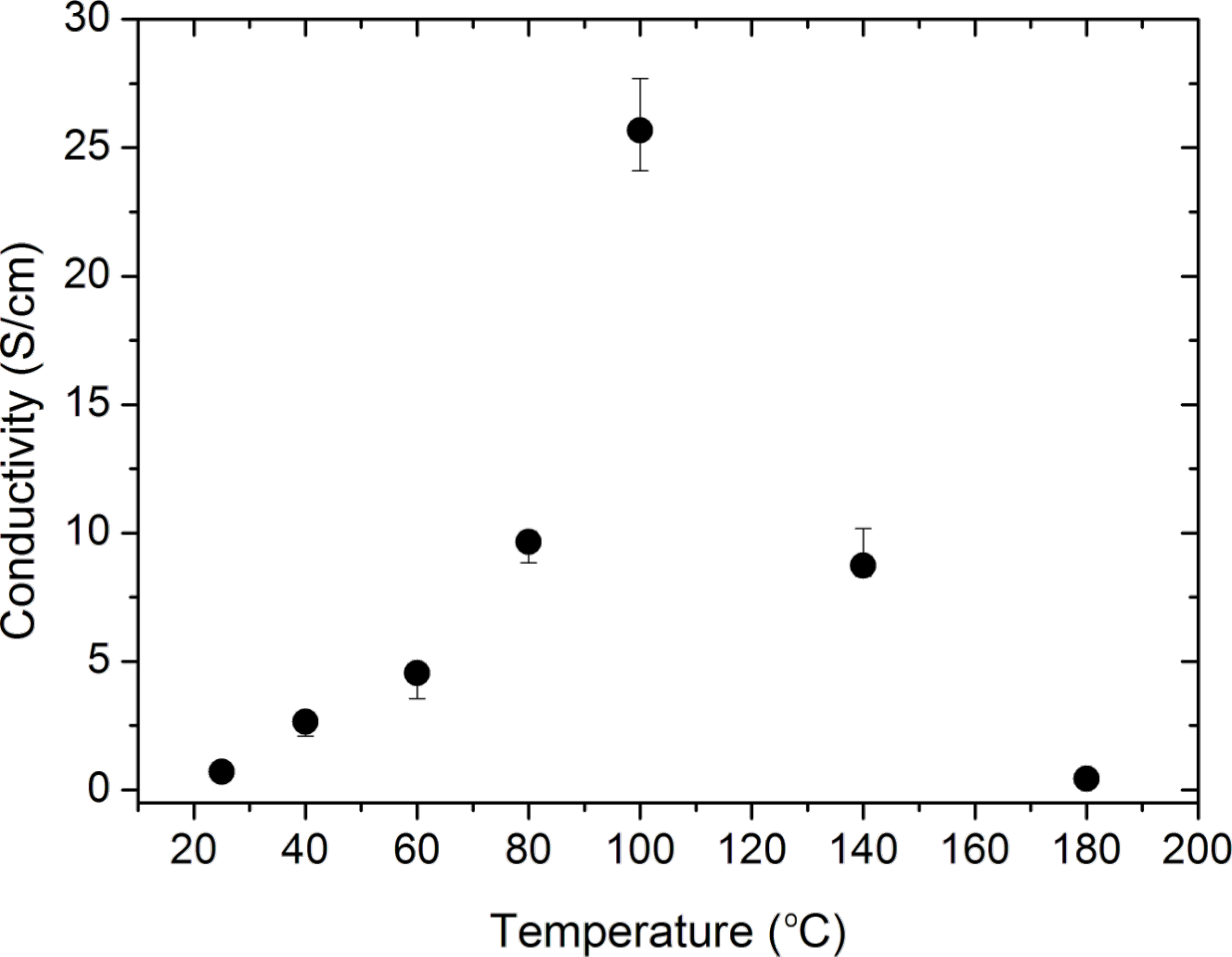
Electrical conductivity of PANI thin films at different annealing temperatures. The conductivity of the films increases with increasing annealing temperatures up to 100 °C because of the increase in crystallinity. Above 100 °C the crystalline structure is damaged leading to the reduction of conductivity.

The conductivity studies of the thin film samples were performed with a four-point probe in air. Figure 5 shows the conductivity values of the as-deposited (25 °C) samples and samples annealed at temperatures ranging from 40 to 180 °C for 4 h. The highest conductivity value of ca. 26 S/cm was obtained with the sample annealed at 100 °C. This conductivity increase is attributed to the increase in crystallinity with annealing, which leads to reduced hopping distance between chains and crystal domains [50]. However, above 100 °C the polymer starts degrading, resulting in damage to the crystalline structure and reduction of the conductivity.

**Figure 6:**
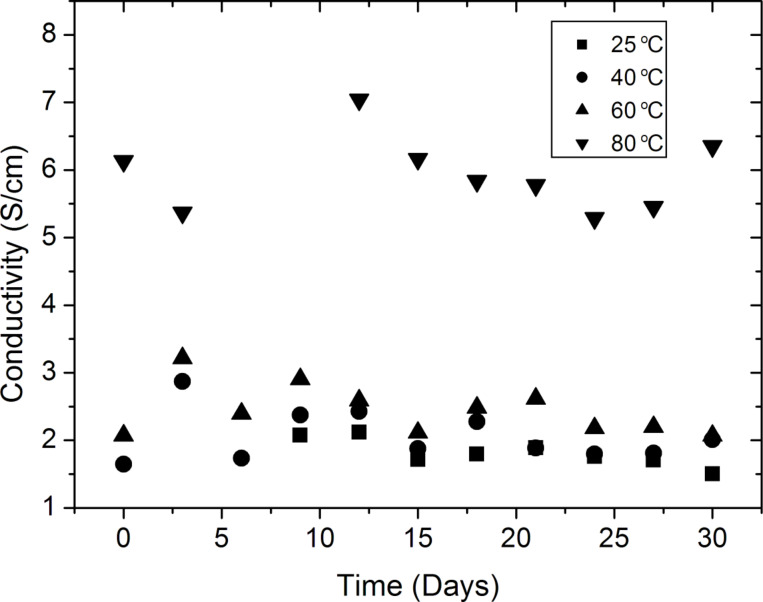
Time dependence of the electrical conductivity of PANI thin films annealed at different temperatures. The decrease in the conductivity after 30 days was less than 2% and did not depend on the annealing temperature.

For sensor applications, the long-term stability of the deposited films was investigated. The conductivities of the PANI coated glass were recorded with a four-point probe in air over 30 days. Figure 6 shows that the decrease in the conductivities of the samples was less than 2% at the end of 30 days, indicating the electrical stability of the films required for long-term applications. Furthermore, the electrical stability of the samples was observed to be independent of the annealing temperatures.

For humidity experiments, circular gold electrodes were evaporated on the PANI films and the resistance was measured using a two-point probe to ensure consistency with the electrical characterization of the nanotube samples. The diameters of the gold electrodes were optimized at 200 μm in order to provide better DC resistance. The lower resistance of 3772 kΩ of PANI-coated glass with electrodes compared to the resistance of 65664 kΩ of PANI-coated glass without electrodes measured in air can be explained by the reduced contact resistance between the PANI thin film and the probes in the presence of gold electrodes.

**Figure 7:**
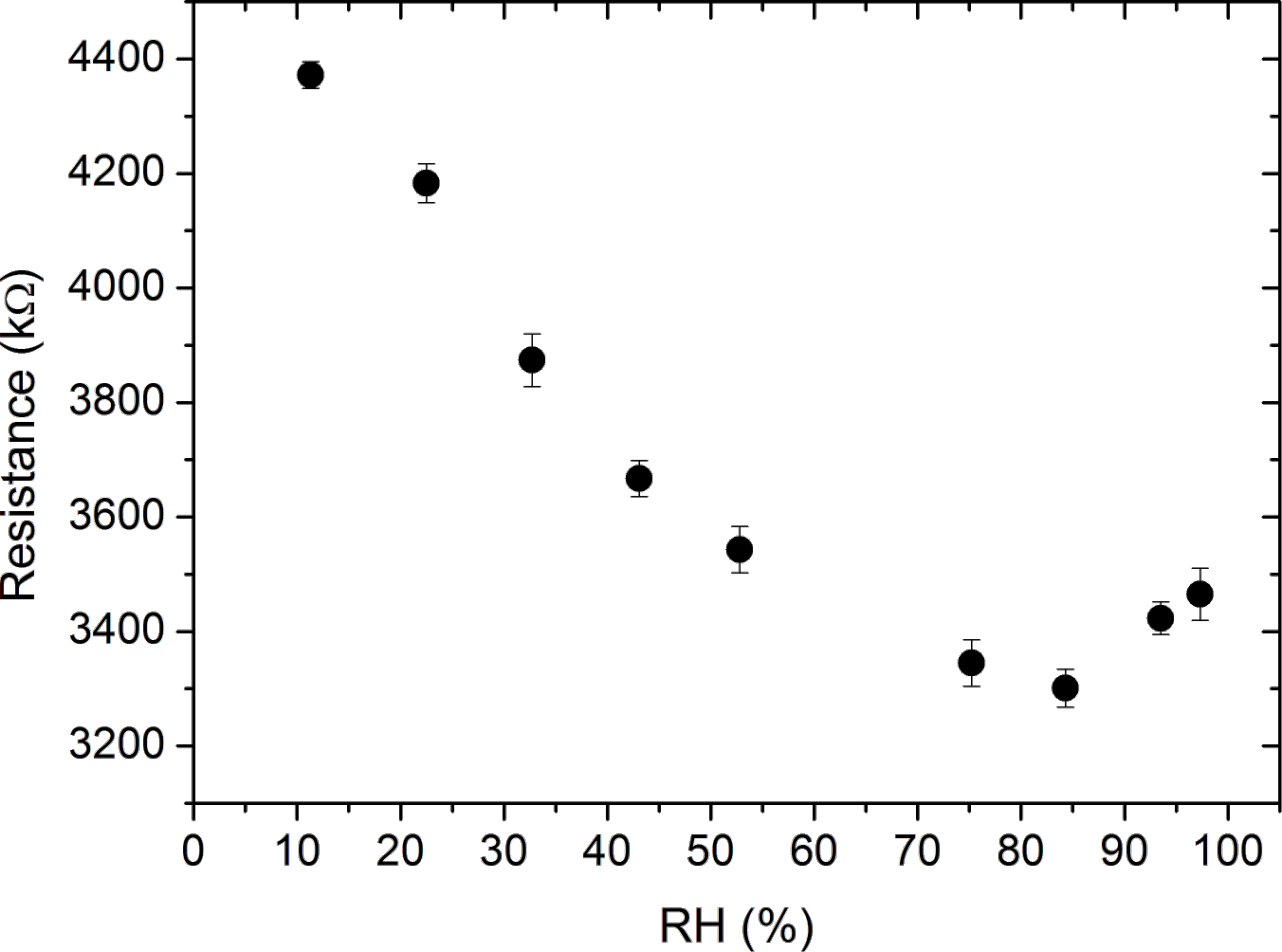
Resistance of PANI thin films as a function of the relative humidity measured using a two-point probe. The parabolic dependency is the result of the competitive effects of doping and swelling of the polymer.

For PANI flat films, the actual resistance values (*R*) versus relative humidity (RH%) are plotted in Figure 7. The resistance slightly decreases as RH% increases up to a certain value (RH% of 84.3%). Above 84.3% resistance starts to increase with humidity. The change in the conductivity of the PANI polymer with humidity is the result of the increasing doping level of the polymer due to the proton exchange facilitated by the H-bonds between the water molecules and N-atoms in the backbone [51]. The ionizable water molecules dissociate into positive protons and negative hydroxyl ions upon entering the polymer chain. The protons dope the polymer further until the undoped parts of the emeraldine salt is mostly doped with the H^+^ ions, after which swelling starts dominating. Swelling of the polymer due to excess water in the ambient results in higher hoping distances and creates distortion in the polymer chains, reducing the conductivity. The maximum resistance of 4372 kΩ was obtained at RH% of 11.3%, whereas the minimum was obtained at 84.3% which is 3301 kΩ.

**Figure 8:**
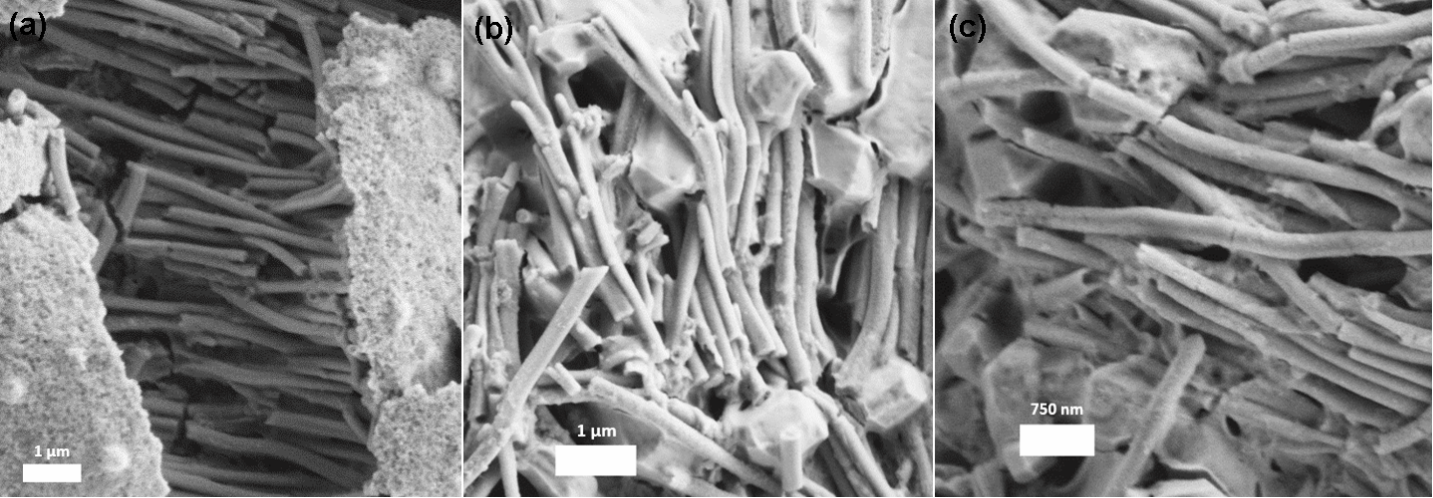
High resolution SEM images of (a) coaxial PANI/pHEMA and (b-c) PANI single component nanotubes.

Single-component PANI and coaxial PANI/pHEMA nanotubes were fabricated using sacrificial AAO membranes. The same process parameters during the polymer deposition were used during the fabrication of both nanotubes and thin films. Figure 8 shows the SEM images of the coaxial and single component PANI nanotubes after removal of the AAO membrane. The fabricated nanotubes are approximately 200 ± 10 nm in diameter with lengths of 3–4 μm.

**Figure 9:**
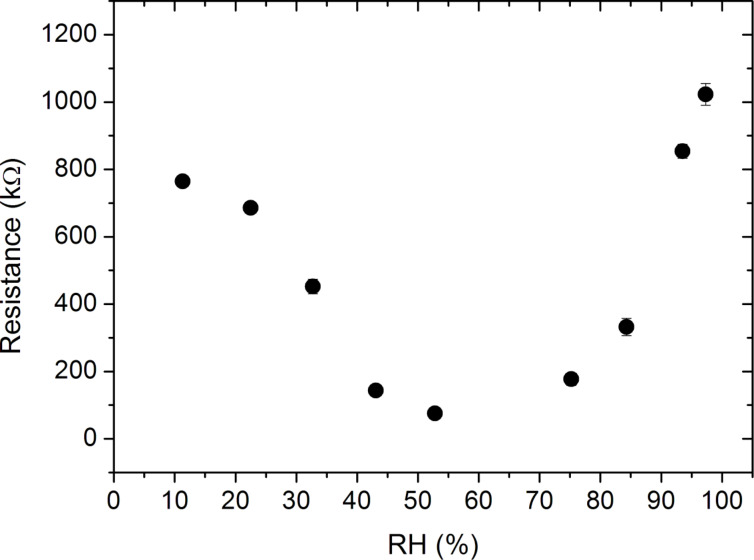
Resistance of single-component PANI nanotubes as a function of the relative humidity. The competing effects of doping and swelling lead to the parabolic behavior, which is similar to the observed behavior in PANI thin films.

Circular gold electrodes were evaporated on the nanotubes for the resistance measurements and the change in the resistance of the nanotubes with humidity was measured using two-point probe. For pure PANI nanotubes the resistance at different relative humidity values is shown in Figure 9. The maximum resistance, which is obtained at 97.3%, is 1023 kΩ while the minimum resistance was measured as 75 kΩ at 52.8%. It should be noted that since the measurements are taken 30 s after the samples are removed from the humid environment, the measured resistance values may be smaller than the resistances when the samples are in the humid atmosphere. However, the trend of the resistance change with the humidity is not expected to be affected by this delay.

The significant resistance difference between PANI nanotubes and PANI thin film stems from the alignment of the polymer chains when deposited inside the pores of a template [36]. This supermolecular order leads to improved conjugation lengths with fewer bends and kinks in the linear polymer chains [52] leading to increased conductivities in nanotubes compared to thin films.

A similar parabolic dependence of resistance on the humidity as observed in the thin films was also observed in the nanotube samples (Figure 9). This type of parabolic dependence of resistance on the humidity was previously reported for PANI nanotubes [53]. The competition between the doping and swelling effects determines at which RH% value the reversal in the behavior of resistance will occur [33]. Comparing the RH% value at which this reversal occurs for the PANI thin films to that of the nanotubes, it is observed that for PANI nanotubes this transition occurs at 52.8%. This is significantly lower than 84.3% that is observed for thin films. This difference can be explained by the high surface-to-volume ratios of the nanotubes compared to the thin films. Due to their high surface-to-volume ratios, more polymer chains are exposed to water molecules in the nanotubes. Even lower humidity levels enable the undoped regions of polymer chains of the nanotubes to be doped with H^+^ ions, resulting in an early onset of this transition behavior.

Comparing PANI thin films (Figure 7) to the nanotubes (Figure 9) in terms of the dependence of their resistance on relative humidity, it is observed that the dependence of resistance on RH% is significantly stronger for nanotubes compared to the thin films. The resistance of nanotubes changes from 1023 kΩ to 75 kΩ whereas for the thin films, the change of resistance is only 25%. The higher sensitivity of the nanotubes to the changes in relative humidity can again be explained by the high surface-to-volume ratio of the nanotubes, enabling more water molecules to interact with polymer chains. This improved sensitivity is especially desirable for humidity sensors.

**Figure 10:**
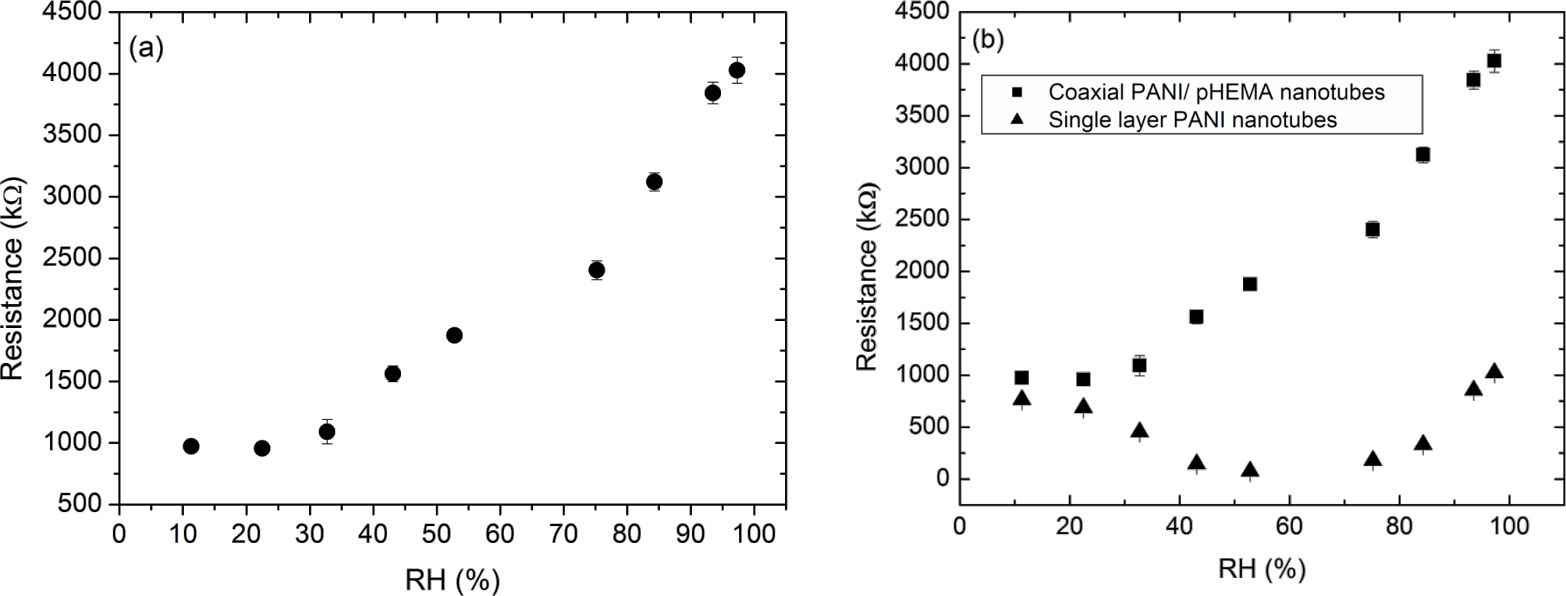
(a) Change in the resistance of coaxial PANI/pHEMA nanotubes with relative humidity. (b) Comparison of the single component and coaxial nanotubes in terms of humidity effect on resistance. The parabolic behavior observed in single-component nanotubes is not observed in the coaxial nanotubes, suggesting the dominance of the swelling effect in the presence of pHEMA hydrogel inner layer.

The main issue with the nanotubes sensor is that within the measurement range of 0–100% relative humidity, the resistance values go through a minimum, resulting in same resistance values at two different humidity levels [33]. As explained in the earlier sections, this minimum is due to the competition between the swelling and doping effects. Incorporating another polymer layer that is sensitive purely to humidity is expected to distinguish between low and high humidity levels, causing differences in the measured resistance values. For this purpose, coaxial nanotubes with pHEMA inner layers and PANI outer layers were fabricated. pHEMA is an insulating hydrogel that is highly sensitive to ambient humidity. The effect of relative humidity on the resistance changes for the coaxial nanotubes is plotted in Figure 10a. According to these results, the resistance values increase parabolically with increasing humidity levels. The increase of resistance is limited (5%) between the humidity levels of 11.3% and 32.7%, indicating poor sensitivity, which is not desirable for a sensor. However, above 32.7% resistance values increase significantly until 97.3% of relative humidity. The maximum resistance of 4027 kΩ was obtained at 97.3%, whereas the minimum resistance was 957 kΩ at the humidity level of 22.5%.

Figure 10b compares coaxial nanotubes to the single-component nanotubes in terms of the dependence of their resistance on relative humidity. The significant difference between the single-component and the coaxial nanotubes in terms of the resistance dependence on the humidity levels stems from the existence of hydrogel pHEMA in the coaxial nanotubes. As stated earlier, pHEMA is sensitive to humidity and swells or shrinks in response to the water level in the ambient. As humidity increases, pHEMA swells which results in an increased distance between each polymer chain, affecting the electronic structure of the nanotubes. The swelling of the inner pHEMA layer, leads to an overall increase in the nanotube diameter, and thus swelling of the outer PANI layer. The longer distances between PANI chains increase the hoping resistance of polymer structure so that the resistance of coaxial nanotubes increases with increasing humidity levels. The doping effect, therefore, is dominated by the swelling effect in the presence of pHEMA layer. At low humidity levels (up to 22.5%), on the other hand, due to the limited swelling of the pHEMA layer, doping effect balances out the swelling effect, resulting in weaker dependence of resistance on humidity.

**Figure 11:**
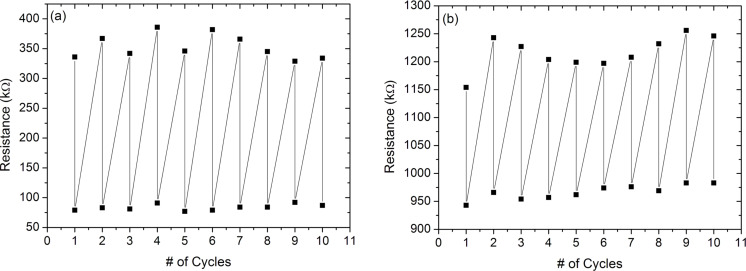
Cyclic resistance measurements of (a) the single component PANI nanotubes at RH% of 35% and 52.8% and (b) coaxial PANI/pHEMA nanotubes at RH% of 35% and 22.5%. At the end of 10 cycles, the change in the resistance is less than 10%.

The stability of single-component and coaxial PANI nanotubes sensors was studied by performing cyclic measurements at two different RH% values. Figure 11a shows the measured resistance of the single-component nanotubes at RH% values of 35% and 52.8%. In the first cycle, the resistance was measured as 336 kΩ and 79 kΩ at 35% and 52.8%, respectively, whereas, in the tenth cycle, the PANI resistance was 334 kΩ and 87 kΩ at 35% and 52.8%, respectively. The change in measured resistance values at both humidity levels is lower than 10%, indicating the stability of the sensors at the end of 10 cycles. The cyclic measurements of coaxial nanotubes were repeated 10 times at RH% values of (35%) and 22.5% (Figure 11(b)). In the first cycle, the resistance was measured as 1154 kΩ and 943 kΩ at 35% and 22.5%, respectively. In the tenth cycle, the resistance was 1246 kΩ and 983 kΩ, at 35% and 22.5%, respectively. The change in the resistance values at the end of 10 cycles is less than 10%, confirming the stability of the nanotubes.

## Conclusion

Single-component conducting PANI nanotubes and coaxial polymer nanotubes with PANI outer layer and hydrogel pHEMA inner layer were synthesized using iCVD and oCVD techniques. Ability to control the thickness during deposition via these vapor-phase methods allowed depositing two layers of different polymers inside the pores of AAO track-etch membranes.

The characterization of the PANI thin films deposited by the vapor-phase oCVD method showed that crystalline PANI thin films could be obtained upon annealing the samples. Conductivity also increased with annealing temperature due to the reduced hopping distance between chains and crystal domains. The results obtained agree with the conventional, solution-based PANI deposition methods reported in literature.

Performance of the single-component and coaxial nanotubes as humidity sensors were tested under different humidity conditions. The competition between the doping and swelling mechanisms of the polymer upon exposure to humidity leads to an increase in the conductivity with humidity until a specific humidity level, above which conductivity decreases. To improve the sensitivity of the sensors an inner hydrogel layer was introduced, delaying the response of the PANI layer. The hydrogel layer in the coaxial nanotubes might further facilitate the swelling effect, which dominates doping, resulting in resistance values that continuously increase with humidity.

The ability to tune the response of the nanotube sensors to humidity by introducing a hydrogel layer will help to improve the sensitivity of the sensors. Furthermore, by incorporating different polymers in the coaxial sensors application areas of these sensors can be extended.

## Experimental

The monomers aniline (99.5%, Sigma-Aldrich), HEMA (99%, Sigma Aldrich), the crosslinker ethylene glycol dimethacrylate (98%, Sigma Aldrich) (EGDMA), the initiator *tert*-butyl peroxide (98%, Sigma Aldrich) (TBPO) and the oxidant antimony pentachloride (99%, Sigma Aldrich) (SbCl_5_) were used as received.

Aniline was heated in a metal jar up to 60 °C while SbCl_5_ was kept at room temperature in a glass jar. Both chemicals were delivered to the system in vapor phase through different ports facing the substrate surface. Glass slides, Si(100) wafers and anodic aluminum oxide template (AAO) with pore sizes of 200 nm were used as substrates and were coated simultaneously. Glass slides were used for electrical conductivity measurements and UV–visible spectroscopy (UV–vis). Si wafers were used for Fourier-transform infrared (FTIR) spectrophotometry, Raman spectroscopy, atomic force microscopy (AFM) and electron microscopy. AAO templates were used for PANI nanotube synthesis. During deposition, the flowrates of aniline and SbCl_5_ were maintained at 1.6 sccm and 1.2 sccm, respectively, at 25 mTorr of operating pressure for 15 min. The stage temperatures varied between 25 and 80 °C for different experiments. After the polymer coating, PANI-coated Si wafers were annealed at temperatures ranging between 40 and 180 °C for 4 h in a vacuum oven.

For PANI flat thin film characterization, FTIR spectrophotometry (Thermo Fischer Scientific Model NICOLETiS10) and Raman spectrometry (Renishaw, inVia Reflex) were used to analyze chemical properties of PANI. FTIR spectra were acquired with 4 cm^−1^ resolution. Raman measurements were carried out at 532 nm wavelength and 50 mW power. Furthermore, UV–vis spectrometry (Shimadzu, UV-VIS 3150) was used on PANI-coated glass slides in order to find the band gap of fabricated PANI and to confirm the electrical conductivity of the film by calculating band-transition energies. Atomic force microscopy (Bruker Multimode 8, ScanAsyst) was used to acquire topography and surface roughness of as-deposited and annealed PANI samples.

X-ray diffraction (XRD) (Bruker, D8 Advance XRD) analysis was performed to study the crystalline state of the annealed and as-deposited PANI films. The measurements were taken on as-deposited PANI and PANI samples annealed at 80 °C at 2θ angles of 5–40° in order to eliminate the peak originating from the Si(100) planes. Each measurement took 4 h for an adequate signal-to-noise ratio. Thickness of the films on Si wafer and glass substrates was measured with a spectroscopic ellipsometer (M-2000, J. A. Woollam) at 65, 70, and 75° within a range of 300–800 nm. For electrical characterization of the PANI thin films, *I*–*V* curves were obtained using a four-point probe at 0.01 μA and the measurements were taken at four different locations (Lucas Labs Pro 4, Keithley 2400 Sourcemeter).

**Figure 12:**
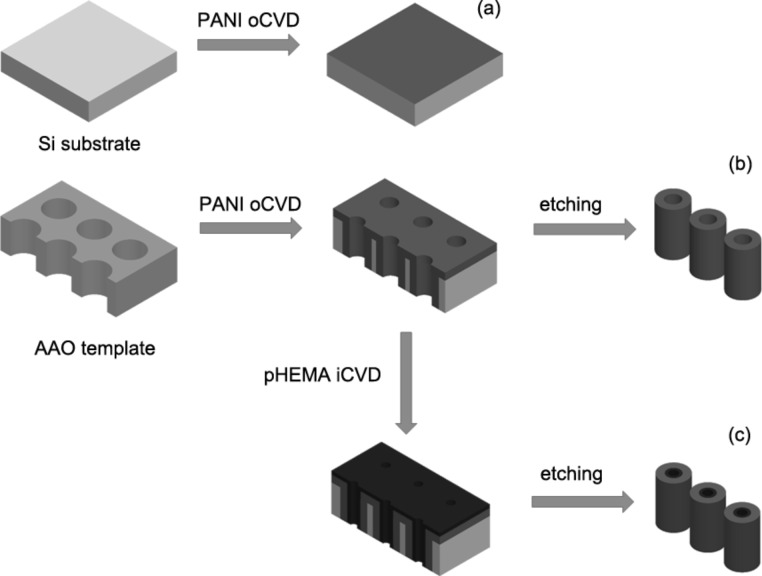
Fabrication steps of nanostructures. (a) PANI thin films are prepared by coating thin layer of PANI on Si surfaces using oCVD. (b) Single-component PANI nanotubes are fabricated by coating the pores of templates with PANI using oCVD. The templates are then etched to release the nanotubes. (c) Coaxial PANI/pHEMA nanotubes are prepared by first coating the pores of templates with PANI polymer using oCVD, followed by iCVD coating of the pores with pHEMA. As the final step the templates are etched to release the nanotubes.

For the fabrication of the coaxial PANI/pHEMA nanotubes PANI was first deposited on AAO templates via oCVD and then the coated templates were exposed to oxygen plasma to remove excess polymer layer on top of AAO membranes. Subsequently, PANI-coated templates were put inside an iCVD chamber for pHEMA deposition. During iCVD depositions, HEMA and EGDMA were heated up to 70 and 85°C, respectively, and TBPO was kept at room temperature. The deposition was performed at 120 mTorr with a stage temperature of 40 °C. The flowrates of HEMA, EGDMA and TBPO were set to 0.8, 0.11 and 1 sccm, respectively. After the deposition, AAO templates coated with PANI and pHEMA were exposed to oxygen plasma (Torr) at 50 W to remove the excess film on the top of the templates. Afterwards, AAO templates were attached to Si wafers and immersed in 0.5 M HCl solution for 48 h to release the coaxial nanotubes, which were then allowed to dry in the air for two days. This allowed immobilization of the free-standing nanotubes on Si wafers for imaging and sensor studies. Figure 12 shows the fabrication steps of nanostructures used in this study. The synthesized coaxial nanotubes had PANI on the outer side and pHEMA inside. Images of the nanotubes were taken with a field-emission scanning electron microscope (FESEM, Zeiss, SUPRA VP 35).

For the electrical characterization of the PANI thin films and PANI/pHEMA nanotubes, an array of chrome (3 nm) and gold (150 nm) electrodes with a diameter of 200 µm and a spacing of 200 µm were deposited on the nanotubes and thin films using an e-beam evaporator (Torr). Prior to the e-beam evaporation, conventional lithography with a shadow mask was used to create a pattern for the electrodes. The photoresist AZ 5214 E (Merck GmbH), the developer AZ 726 (MIF) (Merck GmbH) and deionized water (stopper) were used for the lithography. After the e-beam evaporation, samples were annealed at 100 °C for 4 h. Optical microscope (Zeiss, Axio Scope A1 MAT) image was taken to show the gold electrodes on the PANI thin film surface.

Both thin film and nanotube samples were then tested for sensor performance. For this purpose, a 2-Point probe (Keithley, 2401 Sourcemeter) was used for resistance measurements with constant DC current of 1 μA. In order to vary ambient humidity, several saturated salt solutions with different humidity levels were prepared in DI water. Table 1 lists the salt solutions and their relative humidity (RH) at room temperature.

**Table 1:** Salt solutions and the relative humidity levels obtained in a sealed box by using these solutions.

salt solution	LiCl	CH_3_CO_2_K	MgCl_2_	K_2_CO_3_	Mg(NO_3_)_2_

relative humidity	11.3%	22.5%	32.7%	43.1%	52.8%

salt solution	NaCl	KCl	KNO_3_	K_2_SO_4_	

relative humidity	75.2%	84.3%	93.5%	97.3%	

**Figure 13:**
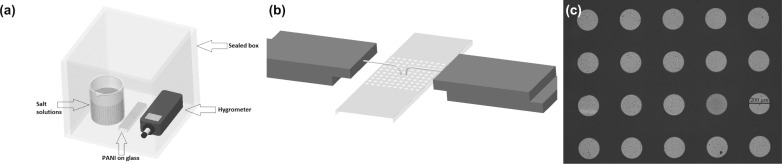
Experimental setup of humidity sensor measurements. (a) The sealed box containing the salt solution, the hygrometer and the nanotube sample. (b) The two-point probe station used for the resistance measurements. The probe station is outside the sealed box. (c) Optical microscope image of the gold electrodes.

Each salt solution and the samples were placed in a sealed box to isolate them from air (Figure 13) For resistance measurements, the samples were taken out from the sealed box and put on the two-point probe station. The initial measurements were taken 30 s after removing the samples from the sealed box. The cyclic resistance measurements were done by leaving the sample in ambient air (35% RH) for 10 min then measuring the resistance of the sample and repeating the same process for 52.8% RH environment within a sealed box.
